# Tetrahydrobiopterin Improves Endothelial Function in Cardiovascular Disease: A Systematic Review

**DOI:** 10.1155/2014/850312

**Published:** 2014-12-04

**Authors:** Qiongying Wang, Mina Yang, Han Xu, Jing Yu

**Affiliations:** Department of Cardiology, The Second Hospital of Lanzhou University, 82 Cuiyingmen Street, Lanzhou, Gansu 730030, China

## Abstract

*Background*. Tetrahydrobiopterin (BH_4_) is a cofactor of nitric oxide synthase (NOS). Nitric oxide (NO) bioavailability is reduced during the early stage of vascular diseases, such as coronary artery disease, hypercholesterolemia, hypertension, and diabetic vasculopathy, and even throughout the entire progression of atherosclerosis. *Methods*. A literature search was performed using electronic databases (up to January 31, 2014), including MEDLINE, EMBASE, and Cochrane Central Register of Controlled Trials (CENTRAL), using an established strategy. *Results*. Fourteen articles were selected with a total of 370 patients. Ten of the fourteen studies showed a significant improvement in the endothelial dysfunction of various cardiovascular disease groups with BH_4_ supplementation compared with the control groups or placebos. Three studies showed no positive outcome, and one study showed that low-dose BH_4_ had no effect but that high-dose BH_4_ did have a significantly different result. *Conclusions*. This review concludes that supplementation with BH_4_ and/or augmentation of the endogenous levels of BH_4_ will be a novel approach to improve the endothelial dysfunction observed in various cardiovascular diseases. BH_4_ might be considered to be a new therapeutic agent to prevent the initiation and progression of cardiovascular disease.

## 1. Introduction

Cardiovascular diseases (CVDs), such as coronary artery disease, hypercholesterolemia, diabetes, hypertension, and stroke, remain the largest cause of mortality and morbidity in the world. In 2014, the attributable fractions of adjusted estimated population for the mortality of CVDs are as follows: 40.6% for high blood pressure, 13.7% for smoking, 13.2% for poor diet, 11.9% for insufficient physical activity, and 8.8% for abnormal blood glucose levels [1]. Abnormal endothelial function appeared as an early feature of all CVDs and risk factor syndromes, resulting in the loss of normal homoeostatic pathways that act to inhibit disease processes such as inflammation, thrombosis, and oxidative stress [2, 3].

The endothelium is the largest endocrine organ in the human body and can be involved in the control of vascular tone, platelet reactivity, coagulation, and permeability [4]. Thus, healthy endothelium can protect against excessive/abnormal inflammation and coagulation [5], which are the key processes in CVD development and progression. Furthermore, endothelial function was demonstrated to serve as a predictor of cardiovascular events [6, 7]. Therefore, the evaluation of endothelial function is vital to generate and determine a more effective or final therapeutic strategy for cardiovascular diseases. From a pathophysiologic standpoint, there is an important focus on the prevention and treatment of vascular diseases via the restoration of the normal biosynthesis of nitric oxide (NO) and the reduction of the excessive generation of superoxide anions and reactive oxygen species (ROS).

Tetrahydrobiopterin (BH_4_) is an essential cofactor for a set of enzymes that are of pivotal metabolic importance, including four aromatic amino acid hydroxylases (AAAH), three nitric oxide synthases (NOS), and alkylglycerol monooxygenase (AGMO). Phenylalanine hydroxylase (PAH) was the first enzyme recognized to depend on BH_4_ [8]. Phenylketonuria is a genetic disorder characterized by a deficiency of PAH; BH_4_ may provide good phenylalanine control in the patients who respond to oral administration of BH_4_. NOS is a critical enzyme militated in the production of the messenger molecule NO, which is generated from L-arginine. BH_4_ is inseparably considered to be a cofactor of NOS enzymes for the progression of NO synthesis [9, 10]. When BH_4_ is limited, under the conditions of oxidative stress, BH_4_ can be readily oxidized to dihydrobiopterin (BH_2_) and eventually converted into biopterin, especially when NOS cofactor activity is not needed. When NOS is uncoupled, ROS rather than NO is produced. NO is used as a soluble gas continuously synthesized from the amino acid L-arginine in endothelial cells via the constitutive calcium-calmodulin-dependent enzyme NOS. This substance has a wide variety of biological properties that maintain vascular homeostasis, including the modulation of vascular dilator tone, regulation of local cell growth, and protection of the vessel from the injurious consequences of platelets and cells circulating in the blood. In the early period of different CVDs, the bioavailability of NO is reduced. In humans, endothelial function is altered in different subjects with vascular disease status and correlated with risk factor profiles [2, 11]. Importantly, several prospective studies have identified that supplementation with BH_4_ improves endothelial function in patients with coronary artery disease, hypercholesterolemia, hypertension, and diabetic vasculopathy [12–15]. Moreover, there are published clinical studies of BH_4_ therapy in the pathogenesis of other vascular diseases, such as pulmonary hypertension [16] and smoking [17, 18], as well as in aging [19–21].

In this regard, supplementation with BH_4_ and/or strategies that augment the endogenous levels of BH_4_ have been recently identified to be novel approaches that can exert salutary effects on the endothelial dysfunction induced by a variety of vascular diseases. This concept and its therapeutic implications are the focus of considerable investigation, which will likely generate an enlarged spectrum of therapeutic agents available for CVDs.

## 2. Materials and Methods

A systematic review of the literature concerning BH_4_ to improve vascular endothelial function in adult patients was conducted using the recommended guidelines provided by the Cochrane Handbook for the Systematic Reviews of Interventions.

### 2.1. Search Methods for the Identification of Studies

To select eligible studies, a search was performed of electronic databases, including PUBMED, MEDLINE, and the Cochrane Library, using a search strategy that depended on combinations of the keywords tetrahydrobiopterin/BH_4_ and endothelial function or endothelial dysfunction. The last search was updated to January 31, 2014. We deliberately broadened the search to ensure the inclusion of all relevant articles. All the bibliographies of papers retrieved from the search were also screened for additional articles. Only full publications in peer-reviewed journals were selected for potential inclusion in the review.

### 2.2. Study Selection Criteria

Two reviewers independently assessed titles, abstracts, and/or the full-text papers of the records retrieved from the electronic database searches for possible inclusion according to the predefined selection criteria: (1) type of study, only RCTs were selected for further assessment; (2) participants, only CVD patients older than 18 years were included, regardless of gender; (3) type of intervention, the intervention used any generation of BH_4_, alone or combined with other substances, irrespective of the administration approach, and the intervention in the control group was a placebo, alone or combined with other substances; and (4) outcomes, trials focused on the effect of supplementation of BH_4_ on endothelial function in patients with CVD.

### 2.3. Quality Assessment

This study is a “qualitative systematic review” without a meta-analysis. The methodological quality of the RCTs was assessed independently by two reviewers (see Table 1) according to the methods recommended in Section Six of the Cochrane Handbook for Systematic Reviews of Interventions, Version 5.1.0.

## 3. Results

### 3.1. Description of Selected Studies

**Figure 1:**
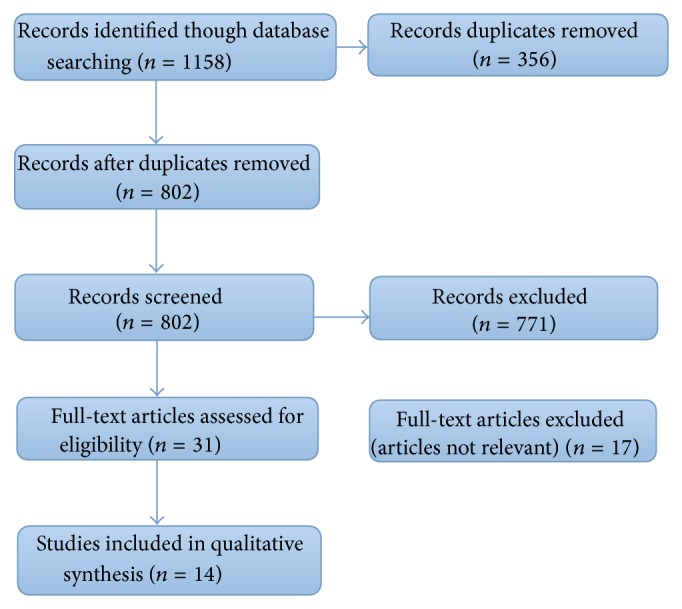
Flow of search.

Citations and abstracts were downloaded into Mendeley and Endnote 6 by independent researchers, and any duplicates were deleted. The main search strategy identified 802 publications, and 356 were excluded because of duplication (Figure 1).

A preliminary screening of the titles and abstracts was performed according to the following inclusion criteria: studies related to BH_4_ and endothelial function. We excluded reviews, meeting notes, book chapters, animal experiments employing qualitative methods, findings derived from qualitative methods, interviews, and observations or participant observations. Access to the full text of the remaining articles was then sought (see Figure 1).

### 3.2. Tetrahydrobiopterin in the Treatment of Cardiovascular Disease

In a range of in vivo pharmacological experiments, clinical studies have been employed to explore the role of BH_4_ on eNOS function in the context of cardiovascular diseases, including coronary artery disease, hypercholesterolemia, hypertension, and diabetic vasculopathy. Based on the resulting experimental evidence, endothelial BH_4_ bioavailability has emerged as a rational therapeutic target in vascular disease states (see Table 2).

### 3.3. Coronary Artery Disease

BH_4_ was administered acutely or on a short-term basis, delivered via intracoronary/intra-arterial infusion [13, 22–25]. In one study, oral BH_4_ was used at a low dose (400 mg/d) or high dose (700 mg/d) for 2 to 6 weeks [26]. In another study, BH_4_ alone did not influence the vessel area but did prevent vasoconstriction in response to acetylcholine (ACh) (+2 ± 3%, NS, versus baseline) in 15 of the patients with endothelial dysfunction in the trial. Correspondingly, calculated volume flow showed the highest value after coinfusion with Ach and BH_4_ [25]. BH_4_ significantly improved acetylcholine-induced increases in coronary blood flow (CBF) in patients with diminished flow responses but exerted no effect in those with normal flow responses [22]. However, no difference was observed in the Ach response due to the coinfusion of BH_4_ and Ach with respect to the % change in CBF [24]. Settergren et al. found that the endothelium-dependent vasodilatation was significantly less reduced at 15 and 30 min of reperfusion following L-arginine and BH_4_ infusion than with saline infusion [25]. BH_4_ did not affect the relative changes in the brachial artery diameter from baseline flow-mediated vasodilation (FMD)(%) in type 2 diabetic and coronary heart disease patients [23]. Oral BH_4_ treatment for 2 to 6 weeks significantly augmented the BH_4_ levels in plasma but had no effect on the vascular redox state or endothelial function [26].

### 3.4. Hypercholesterolemia

The method of administration was mainly infusion via the brachial artery [27–29] or coronary ostium [30]. 22 hypercholesterolemic patients were randomized into groups receiving 4 weeks of oral BH_4_ (400 mg twice daily) or placebo [14]. In all studies, BH_4_ restored the vascular function in the patients with hypercholesterolemia. BH_4_ also restored the endothelial function of coronary arteries in the patients with hypercholesterolemia [28, 30]. BH_4_ attenuated the Ach-induced decrease in coronary diameter and restored the Ach-induced increase in coronary blood flow [30]. BH_4_ increased exercise-induced hyperemia in all subjects but had no influence on myocardial blood flow (MBF) at rest or during adenosine-induced hyperemia in all subjects. Flow reserve utilization was increased significantly in hypercholesterolemic subjects but remained unchanged in controls [28]. The vasoconstrictor response to L-monomethyl-arginine (L-NMMA) was significantly increased with BH_4_ treatment compared with saline infusion (*P* < 0.05); additionally, the impaired serotonin-induced vasodilation was restored by this treatment [27]. Localized BH_4_ alone or in combination with other substances augmented the NO-dependent vasodilatation in hypercholesterolemic patients but showed no effect in normocholesterolemic subjects [29]. BH_4_ restored endothelium-dependent, NO-mediated vasodilatation but had no effect on endothelium-independent vasodilatation due to sodium nitroprusside [14].

### 3.5. Hypertension

BH_4_ (500 *μ*g/min) was infused intra-arterially for 5 min. The forearm blood flow (FBF) response to Ach in hypertensive patients increased significantly to the level of normal control subjects [31]. Oral high-dose BH_4_ (400 mg/d) produced a significant decrease in systolic (*P* < 0.03) and mean blood pressure (BP) (*P* < 0.04). The decrease in diastolic BP did not reach statistical significance (*P* < 0.08). No significant change in BP was observed in subjects given low-dose BH_4_ (200 mg/d). There was a significant improvement in FMD with 400 mg of BH_4_ but no significant change with 200 mg of BH_4_ [12].

### 3.6. Diabetic Vasculopathy

In diabetes, cardiovascular disease is a common complication. Endothelial dysfunction occurs as the first step in the pathogenesis of diabetes to promote arteriosclerosis. BH_4_ enhanced vascular response to acetylcholine-induced vasodilation, whereas endothelium-independent vasodilation was not affected in diabetes patients [15, 25]. In contrast, Cosentino et al. found that BH_4_ improved glucose disposal in individuals with type 2 diabetes but without any discernible changes in vasodilation or macrovascular blood flow [14]. BH_4_ restored the endothelium-dependent vasodilation induced by an oral glucose challenge in the forearm of healthy subjects [32].

## 4. Discussion

Previous research has indicated that maintaining adequate BH_4_ levels in the endothelium is critical in regulating the balance of NO and superoxide synthesis in CVDs. Numerous studies have examined the effect of BH_4_ supplementation on endothelial dysfunction in a wide variety of CVDs, including coronary artery disease, hypercholesterolemia, hypertension, and diabetic vasculopathy. The substitution of BH_4_, an essential cofactor of NOS and a scavenger of oxygen-derived free radicals, is able to restore coronary vasomotion in response to Ach [13, 22, 30]. Supplementation with BH_4_ augments forearm vessel endothelium-dependent vasodilation by improving endothelial dysfunction [25, 27, 31]. The flow reserve utilization of the coronary microcirculation in hypercholesterolemic subjects is significantly reduced but is nearly restored after BH_4_ infusion [28]. BH_4_ augmented NO-dependent vasodilatation during local heating by increasing the plateau in skin blood flow in hypercholesterolemic humans [29].

These studies involved a limited number of patients in whom BH_4_ was administered acutely or on a short-term basis, and BH_4_ was typically delivered via intracoronary/intra-arterial infusion, which is not representative of a suitable route of administration for chronic disease management. The breadth of preclinical and acute clinical data implicating BH_4_ as a key regulator in endothelial function suggests that oral BH_4_ therapy may be able to prevent or treat CVDs. Clinical trials investigating oral BH_4_ supplementation have shown varied efficacy in numerous disorders with an apparent lack of efficacy in diseases such as hypertension, hypercholesterolemia, and coronary artery disease. Porkert and coworkers showed that oral BH_4_ at a daily dose of 400 mg or higher has a significant and sustained antihypertensive effect in subjects with poorly controlled hypertension but that lower dose (200 mg per day) BH_4_ has no effect [12]. Twenty-two hypercholesterolemic patients were randomized to receive 4 weeks of either oral BH_4_ (400 mg twice daily) or placebo, and age-matched healthy volunteers served as controls. They found that chronic BH_4_ treatment led to an eightfold increase in plasma BH_4_ levels and restored the impairment in endothelium-dependent relaxation due to Ach in hypercholesterolemic patients but did not affect control subjects. Importantly, they also demonstrated that BH_4_ significantly reduced the plasma levels of 8-F2 isoprostane, a marker of oxidative stress, and that the effect of BH_4_ treatment on NO bioavailability is independent of any change in LDL cholesterol [14]. In contrast, oral low-dose (400 mg/d) or high-dose (700 mg/d) BH_4_ for 2 to 6 weeks in patients with established coronary artery disease significantly elevated plasma BH_4_ levels. However, this elevation in plasma BH_4_ was tempered by similar rises in plasma BH_2_ and biopterin, so that the ratio of reduced biopterins to oxidized ones (BH_4_/[BH_2_+biopterin]) in plasma remained unchanged after treatment, with neither molecule having an effect on the vascular redox state or endothelial function [26]. In addition, a phase 2 clinical trial sponsored by the US pharmaceutical company BioMarin failed to observe an ameliorative effect of the oral administration of BH_4_ in patients with poorly controlled hypertension. There are studies providing preliminary evidence that oral BH_4_ could increase artery compliance and decrease arterial stiffness in healthy older men [33] or estrogen-deficient postmenopausal women [34]. 6R-BH_4_ was administered starting at a dose of 2.5 mg/kg and increasing to 20 mg/kg over 8 weeks. This treatment produced an improvement in the 6-minute walking distance, with the most significant improvement at a dose of 5 mg/kg, in patients with pulmonary hypertension [16].

The vascular effects following the oral administration of BH_4_ appear complex and dose dependent, which may be explained by either the rapid clearance of BH_4_ after oral administration and/or an enhanced oxidation to BH_2_, which lacks eNOS cofactor activity. Thus, systemic oxidative stress may play a critical role in determining the degree of oxidation of BH_4_ to BH_2_ and hence the ratio of BH_4_ : BH_2_ and efficacy of the treatment [26]. Recent data from cultured endothelial cells [35, 36] suggest that the intracellular levels of BH_2_ and, more specifically, the ratio between reduced and oxidized biopterins are important in regulating eNOS coupling. Considering that BH_4_ is easily oxidized to BH_2_, strategies should increase the supplementation of BH_4_ with antioxidants. On one hand, they can reduce the oxidation of BH_4_ to BH_2_; on the other hand, they may synergistically decrease oxidative stress and increase nitric oxide. This hypothesis is supported by observations in which the antioxidant vitamin C stimulates eNOS enzymatic activity by increasing the intracellular concentration of BH_4_ [33, 37]. Vitamin C likely exerted its beneficial effects in that study through a variety of molecular mechanisms. In its capacity as an antioxidant, it enhances NO bioavailability by quenching O^2−^, thus limiting the inactivation of NO that occurs when O^2−^ and NO combine to produce OONO^−^ [38]. Vitamin C also stabilizes existing BH_4_ [39] and increases endothelial BH_4_ synthesis [40]. We have demonstrated that plasma biopterin oxidation status is closely linked to the amount of ascorbate in plasma and hence in the diet in vivo [41]. However, studies in larger cohorts of patients would be required to determine whether this dual (BH_4_ plus antioxidant) intervention would be efficacious on a chronic basis.

In addition, acute or short-term supplementation with BH_4_ via intracoronary/intra-arterial infusion has no beneficial effect on endothelial dysfunction in CVDs [23, 24]. These findings suggest that, in humans, BH_4_ does not passively diffuse from the circulating blood into the vascular endothelium. Previous work has indicated that biopterin transport is cell type dependent and that both direct uptake (as BH_4_) and conversion to BH_2_ followed by recycling via dihydrofolate reductase (DHFR) are possible mechanisms [42]. In fact, in patients with coronary artery atherosclerosis, high plasma levels of BH_4_ are associated with low BH_4_ levels in the endothelium [43]. To ensure that BH_4_ is imported into the endothelium, it must undergo oxidation to BH_2_; imported BH_2_ is then regenerated back to BH_4_ by DHFR. A recent study found that human DHFR has very low affinity for 7,8-BH_2_ and that folic acid inhibits 7,8-BH_2_ recycling [44]. Thus, we consider that the low activity of endothelial DHFR is an important factor limiting the benefits of BH_4_ therapies.

## 5. Conclusion

In summary, targeting BH_4_ remains a rational therapeutic strategy in CADs. However, we found that oral BH_4_ treatment in patients with CADs significantly elevates the BH_4_ levels in blood but that this effect is significantly limited by the systemic oxidation of exogenous BH_4_ to BH_2_, which lacks eNOS cofactor activity. More studies should be directed toward interventions that can favorably alter the endogenous BH_4_/BH_2_ ratio in the human vascular endothelium via a selective increase in the absolute BH_4_ levels, the prevention of BH_4_ oxidation, or an increase in BH_4_ recycling. In particular, the effect of antioxidant coadministration to prevent the systemic and vascular oxidation of exogenous BH_4_ warrants further attention. Beneficial effects of acute BH_4_ supplementation on endothelial function have been reported in many human studies. However, the long-term based clinical trials are deficient. Oral administration can be considered to be representative of a suitable administration route for chronic disease management. Therefore, long-term experiments investigating oral BH_4_ supplementation are needed.

## Figures and Tables

**Table 1:** Basic characteristics of the included clinical trials.

Study, year	Methods	BH_4_-treated			Placebo		
		Number	Sex (M/F)	Age	Number	Sex (M/F)	Age
Maier et al., 2000 [13]	Randomized	19	3/16	56 ± 10	∗	∗	∗
Setoguchi et al., 2001 [22]	Randomized	15	10/5	60 ± 11	∗	∗	∗
Nyström et al., 2004 [23]	Randomized; single-blind crossover	6	6/0	59 ± 2	6	6/0	59 ± 2
		5	5/0	57 ± 2	5	5/0	57 ± 2
		5	5/0	29 ± 4	5	5/0	29 ± 4
Worthley et al., 2007 [24]	Randomized controlled	22	4/18	60 ± 9	5	∗	
		25	5/20	60 ± 9	5	∗	
Settergren et al., 2009 [25]	Randomized; blind, crossover	12	12/0	71 ± 1.5	∗		
Cunnington et al., 2012 [26]	Randomized; double-blind; parallel design	30	3/27	∗	19	3/16	68 ± 2
Stroes et al., 1997 [27]	Randomized controlled	13	9/4	32 ± 4	∗		
		13	9/4	28 ± 2	∗		
Fukuda et al., 2002 [30]	Randomized controlled	9	7/2	61 ± 9	∗		
		9	7/2	59 ± 9	∗		
Wyss et al., 2005 [28]	Randomized controlled	9	7/2	54 ± 8	∗	∗	∗
		10	10/0	25 ± 3	∗	∗	∗
Holowatz and Kenney, 2011 [29]	Randomized controlled	9	6/3	53 ± 3	∗		
		9	5/4	49 ± 2	∗		
Cosentino et al., 2008 [14]	Randomized; double-blind; parallel design	11	7/4	61 ± 9	10	10/0	54 ± 10
		9	7/2	54.4 ± 9.5	∗		
Higashi et al., 2002 [31]	Randomized controlled	8	6/2	48 ± 11	∗		
		8	6/2	44 ± 9	∗		
Porkert et al., 2008 [12]	Randomized	24	9/15	∗	∗		
Heitzer et al., 2000 [15]	Randomized controlled	23	7/16	52 ± 2	∗		
		12	8/4	50 ± 3	∗		

**Table 2:** Effects of BH_4_ supplementation in human vascular disease.

Study, year	Disease	Outcome	Administration
Maier et al., 2000 [13]	Ischemia reperfusion injury	Prevents endothelial dysfunction	6R-BH_4_ (Alexis Corp.) intracoronary 10^−2^ M, for 2 min
Setoguchi et al., 2001 [22]	Coronary artery disease	Improves endothelium-dependent vasodilatation	6R-BH_4_ (Clinalfa) intracoronary infusion 4 mg/min for 2 min
Nyström et al., 2004 [23]	Type 2 diabetic and coronary heart disease	Had no effect on endothelial-dependent vasodilation	6R-BH_4_ (Schircks) intra-arterial infusion 500 *μ*g/min
Worthley et al., 2007 [24]	Atherosclerotic disease	Does not improve endothelial function	6R-BH_4_ (Clinalfa) infusion 250 *μ*g/min and 500 *μ*g/min for 6 min
Settergren et al., 2009 [25]	Diabetes (type II) and coronary artery disease	Improves endothelial dysfunction	6R-BH_4_ (Clinalfa) intra-arterial infusion 500 *μ*g/min
Cunnington et al., 2012 [26]	Coronary artery disease	Has no net effect on vascular redox state or endothelial function	6R-BH_4_ (Schircks) 400 mg/d or 700 mg/d per oral for 2 to 6 weeks
Stroes et al., 1997 [27]	Hypercholesterolemia	Restored NO-dependent vasodilatation	6R-BH_4_ (Alexis Corp.) infusion 500 *μ*g/min
Fukuda et al., 2002 [30]	Hypercholesterolemia	Improves coronary endothelial function	6R-BH_4_ (Sigma) intracoronary 1 mg/min for 2 min
Wyss et al., 2005 [28]	Hypercholesterolemia	Restores flow reserve utilization	6R-BH_4_ (Schircks) infusion 10 mg kg^−1^ over 30 min
Holowatz and Kenney, 2011 [29]	Hypercholesterolemia	Augmented NO-dependent vasodilatation	6R-BH_4_ (Sigma) 10 mM
Cosentino et al., 2008 [14]	Hypercholesterolemia	Restores NO bioavailability and endothelial function	6R-BH_4_ (Schircks) 400 mg twice daily orally for 4 weeks
Higashi et al., 2002 [31]	Hypertension	Augments endothelium-dependent vasodilatation	6R-BH_4_ (Sigma) infusion 500 *μ*g/min
Porkert et al., 2008 [12]	Hypertension	Significant improvement in endothelial function in higher doses	6R-BH_4_ (Schircks) oral 5/10 mg kg^−1^ day for 8 weeks and 200/400 mg for 4 weeks
Heitzer et al., 2000 [15]	Diabetes (type II)	Improves endothelium-dependent vasodilatation	6R-BH_4_ (Schircks) intra-arterial infusion 500 *μ*g/min
